# When Thinking Is Outsourced: Cognitive Offloading and the Heterogeneity of Critical Thinking Among Chinese University Students Using Generative Artificial Intelligence

**DOI:** 10.3390/jintelligence14070116

**Published:** 2026-06-24

**Authors:** Shuai Si, Yong Qi, Jingming Xu, Xinyu Qi

**Affiliations:** Beijing University of Civil Engineering and Architecture, Beijing 100044, China

**Keywords:** generative artificial intelligence, cognitive offloading, critical thinking, cognitive autonomy, higher education

## Abstract

Generative artificial intelligence (GAI) enables students to offload cognitive tasks to an external system, yet the consequences of such cognitive offloading for the development of critical thinking—a core dimension of human intelligence—remain underexplored. Drawing upon cognitive offloading theory and distributed cognition theory, this study investigates the heterogeneity of critical thinking outcomes among Chinese university students who use GAI, focusing on how different patterns of human–AI collaboration relate to cognitive autonomy relinquishment. A questionnaire survey was administered to 353 university students across multiple provinces in China. Cluster analysis and regression analysis were employed to identify distinct user profiles and to examine predictors of critical thinking gains and cognitive autonomy. Four distinct user profiles emerged, ranging from “simple Q&A users” (25.2%) to “critical co-thinkers” (15.6%). Learning motivation was the strongest predictor of both critical thinking gains (β = 0.42) and lower cognitive autonomy relinquishment (β = −0.35). Notably, offloading depth positively predicted cognitive autonomy relinquishment (β = 0.25), revealing a paradoxical pattern: sophisticated GAI use was associated with greater dependence. A “high depth–high dependence” subgroup (25.8%) was identified, disproportionately composed of female students and Information and Communication Technology (ICT) majors. The findings challenge the assumption that deeper GAI engagement automatically yields cognitive benefits. Because all constructs were measured through self-report, the findings are interpreted as reflecting students’ perceptions of their cognitive behaviors and abilities; the methodological implications of this design are discussed in detail. Educational interventions should prioritize metacognitive training over technical skill development to ensure that cognitive offloading enhances rather than undermines critical thinking.

## 1. Introduction

The rapid proliferation of generative artificial intelligence (GAI) tools—exemplified by ChatGPT, Claude, and Gemini—has fundamentally transformed how students access information, construct knowledge, and complete academic tasks (Ibrahim et al., 2026). Unlike traditional search engines that retrieve existing information, GAI systems can synthesize novel content, engage in multi-turn dialogue, and assist with complex cognitive tasks ranging from code debugging to essay writing. This technological shift has sparked intense debate among educators and researchers: Does GAI serve as a cognitive amplifier that enhances human thinking, or does it function as a cognitive crutch that undermines the development of critical intellectual capacities?

The concept of cognitive offloading provides a theoretical lens for examining this question. Cognitive offloading refers to the use of external tools or agents to reduce the cognitive demands of a task (Wang, 2026). When students use GAI to generate essay outlines, summarize readings, or solve problems, they are essentially offloading cognitive work to an artificial agent. This process is not inherently detrimental—offloading routine cognitive operations can free mental resources for higher-order thinking. However, the critical question is whether students maintain cognitive agency in this process or gradually cede their thinking autonomy to AI systems.

This study investigates the heterogeneity of critical thinking outcomes among Chinese university students who use GAI. Rather than assuming a uniform effect of GAI use, we examine how different patterns of human–AI collaboration relate to two key outcomes: (a) perceived critical thinking gains, and (b) cognitive autonomy relinquishment. Drawing upon cognitive offloading theory and distributed cognition theory, we develop a conceptual framework that distinguishes between strategic offloading (using GAI as a thinking partner while maintaining cognitive agency) and habitual offloading (using GAI as a thinking replacement). We further explore how individual factors—learning motivation, disciplinary background, and gender—moderate these relationships. The methodological implications of measuring these constructs through self-report instruments are discussed in detail in the Methods and Discussion Sections.

The Chinese context offers a particularly relevant setting for this investigation. Chinese universities have witnessed rapid adoption of GAI tools, with recent surveys indicating that over 70% of university students have used ChatGPT or similar tools for academic purposes (Zhang & Wang, 2025). At the same time, Chinese educational culture has traditionally emphasized rote learning and examination performance, potentially creating conditions where GAI might be used in ways that substitute rather than supplement cognitive effort. Understanding how Chinese students navigate the opportunities and risks of GAI use can inform educational policy and practice both within China and globally.

## 2. Theoretical Framework and Literature Review

### 2.1. Cognitive Offloading Theory

Cognitive offloading theory posits that humans have a natural tendency to reduce cognitive effort by delegating mental operations to external resources (Risko & Gilbert, 2016). This tendency is evolutionarily adaptive—conserving cognitive resources for survival-critical tasks—but becomes problematic when offloading undermines the development of cognitive capacities. The theory distinguishes between two types of offloading: (a) externalizing, which involves storing information externally (e.g., taking notes, using calendars), and (b) delegating, which involves having an external agent perform cognitive work (e.g., asking someone else to solve a problem). GAI represents a particularly powerful form of delegating offloading, as it can perform complex cognitive tasks that previously required human intelligence.

The theoretical foundations of cognitive offloading can be traced to broader debates about the relationship between internal cognition and external resources. Clark and Chalmers (1998) proposed the extended mind thesis, arguing that cognitive processes are not confined within the boundaries of the skull but can extend into the environment through the use of external artifacts. This perspective challenges traditional cognitivist assumptions that mental processes are purely internal and suggests that tools, technologies, and social structures can constitute integral components of cognitive systems. The extended mind thesis provides a philosophical foundation for understanding cognitive offloading not as a mere shortcut or avoidance strategy, but as a fundamental aspect of how human cognition operates in technologically rich environments.

Building on these foundations, Risko and Gilbert (2016) synthesized empirical research on cognitive offloading across multiple domains, identifying key factors that influence offloading decisions. Their work revealed that individuals are more likely to offload cognitive tasks when they perceive the task as difficult, when they have low confidence in their own abilities, and when external resources are readily available and reliable. Importantly, they found that offloading is not inherently detrimental to cognitive performance; rather, its effects depend on whether the offloaded information is later retrieved and integrated into the individual’s knowledge base. This finding suggests that the critical question is not whether offloading occurs, but how it is managed and monitored.

A key insight from cognitive offloading research is that the consequences of offloading depend on how it is deployed. Dunn and Risko (2016) introduced the concept of metacognitive offloading, wherein individuals strategically decide what to offload based on task demands and their own cognitive goals. In their research, the external resources examined were primarily low-level cognitive aids—such as storing information in notes, setting reminders, and delegating simple memory tasks to external devices—rather than the technologically sophisticated tools capable of performing complex cognitive operations that characterize contemporary GAI systems. This distinction is important because the offloading decisions studied by Dunn and Risko involved relatively straightforward choices about information storage and retrieval, whereas offloading decisions in the GAI context involve complex judgments about delegating reasoning, analysis, and creative tasks to an artificial agent. Metacognitive offloaders maintain awareness of what they know and what they have offloaded, and they engage in periodic “cognitive audits” to ensure that offloading serves rather than subverts their learning objectives. In contrast, habitual offloaders develop automatic dependencies on external resources without monitoring the consequences for their cognitive development.

Recent research has begun to examine cognitive offloading in the specific context of AI-mediated learning. A comprehensive review by Wang (2026) found that students who engage in deliberate, metacognitively guided offloading with digital tools demonstrate stronger planning behaviors and better learning outcomes compared to those who offload habitually. Similarly, studies examining AI dependency among high school learners have documented significant negative correlations between uncritical AI reliance and deep learning outcomes (Chiu, 2024). These findings underscore the importance of distinguishing between strategic and habitual offloading patterns when examining the cognitive consequences of GAI use.

The emergence of generative AI has introduced qualitatively new dimensions to cognitive offloading. Unlike traditional external memory aids (e.g., notebooks, calendars) that store information for later retrieval, GAI can generate novel content, synthesize information across domains, and perform complex reasoning tasks. This capability transforms the nature of cognitive offloading from simple information storage to genuine cognitive delegation. As noted by researchers examining AI’s impact on higher-order thinking, the risk is not merely that students will forget information they have offloaded, but that they may fail to develop the cognitive capacities necessary to evaluate, integrate, and build upon AI-generated outputs (Zhao et al., 2025). This concern is particularly acute for critical thinking skills, which require sustained practice in analysis, evaluation, and argumentation—precisely the types of cognitive activities that GAI can now perform on behalf of users.

From a cognitive load theory perspective (Chung et al., 2021), cognitive offloading can be understood as a strategy for managing the limited capacity of working memory. Cognitive load theory distinguishes between three types of cognitive load: intrinsic load (inherent to the task complexity), extraneous load (imposed by instructional design), and germane load (devoted to schema construction and learning). Offloading certain cognitive operations to external resources can reduce extraneous load, freeing working memory capacity for germane processes that support learning. However, this beneficial effect depends critically on what is offloaded: offloading peripheral processing tasks (e.g., calculation, information retrieval) may enhance learning, while offloading core cognitive processes (e.g., analysis, evaluation) may undermine it. The challenge for learners using GAI is to distinguish between these types of tasks and to offload strategically rather than indiscriminately.

### 2.2. Distributed Cognition and Human–AI Collaboration

Distributed cognition theory (Hutchins, 1996) provides a complementary perspective by conceptualizing cognition as a process that extends beyond individual minds to encompass tools, artifacts, and social interactions. From this view, using GAI is not simply “cheating” or “outsourcing” thinking; rather, it represents a new form of distributed cognitive system in which human and artificial intelligence collaborate to accomplish cognitive tasks. The quality of this collaboration depends on how the cognitive workload is distributed between human and AI, and whether the human maintains an active, agentic role in the cognitive process.

Hutchins’s (1996) seminal work on distributed cognition emerged from ethnographic studies of navigation teams on naval vessels, demonstrating how cognitive tasks are accomplished through the coordinated activities of multiple individuals and artifacts. This research revealed that cognitive processes such as problem-solving, memory, and decision-making are not confined to individual minds but are distributed across social and technological systems. The theory emphasizes that the functional unit of analysis for understanding cognition is not the individual but the larger sociotechnical system in which cognitive work occurs. Applied to human–AI collaboration, distributed cognition theory suggests that the relevant question is not whether AI is doing cognitive work, but how the cognitive work is distributed across the human–AI system and whether this distribution supports or undermines the human’s cognitive development.

The distributed cognition perspective has been increasingly applied to understand human–AI interaction in various domains. Research in this area has identified key challenges in maintaining effective cognitive partnerships with AI systems, including issues of trust calibration, situation awareness, and the preservation of human expertise. These findings highlight that effective human–AI collaboration requires not only technical competence in using AI tools but also metacognitive awareness of the division of cognitive labor and the ability to maintain oversight of AI-generated outputs.

Recent research on human–AI collaboration has identified several patterns of interaction. Complementarity occurs when humans and AI contribute different strengths to a task (e.g., AI provides information, human provides judgment). Substitution occurs when AI replaces human cognitive work entirely. Augmentation occurs when AI enhances human cognitive capacities without replacing them. These patterns suggest that the cognitive consequences of GAI use are not uniform but depend on the specific mode of human–AI collaboration adopted by the user.

The concept of cognitive partnership has emerged as a useful framework for understanding productive human–AI collaboration. Rather than viewing AI as either a tool that serves human goals or a competitor that replaces human cognition, the cognitive partnership perspective emphasizes the potential for synergistic relationships in which human and AI capabilities are combined in ways that enhance overall cognitive performance. Research on human–AI partnerships in education suggests that effective partnerships require clear role definitions, mutual transparency about capabilities and limitations between all participants in the learning ecosystem—including educators, students, and AI systems, and mechanisms for maintaining human agency and oversight (Holstein et al., 2019). In this framework, mutual transparency refers to the expectation that all parties—educators who design and scaffold AI-mediated learning activities, students who engage with AI tools, and AI systems whose outputs must be interpretable—should have clear understanding of one another’s capabilities, limitations, and roles in the cognitive partnership. These principles provide guidance for how students might engage with GAI in ways that support rather than undermine their cognitive development.

An important consideration in distributed cognition is the concept of ‘cognitive residue’—the lasting cognitive effects that remain after the external cognitive resource is removed. In traditional distributed cognition research, this concept has been used to understand how working with external tools can shape internal cognitive processes. For example, individuals who regularly use navigation systems may develop different spatial cognition patterns compared to those who navigate independently. Applied to GAI, this concept raises important questions about how sustained use of AI for cognitive tasks may reshape students’ cognitive processes and capabilities. If students consistently offload certain types of cognitive work to AI, they may fail to develop—or may gradually lose—the cognitive capacities required to perform these tasks independently.

### 2.3. Critical Thinking and Cognitive Autonomy

Critical thinking has been defined as “purposeful, self-regulatory judgment which results in interpretation, analysis, evaluation, and inference” (Facione, 1990). It encompasses both cognitive skills (e.g., identifying assumptions, evaluating evidence) and dispositions (e.g., open-mindedness, intellectual humility). In the context of GAI use, critical thinking becomes relevant in two ways: (a) students need critical thinking to evaluate AI-generated content, and (b) GAI use may affect the development of critical thinking capacities.

The conceptualization and assessment of critical thinking has been a central concern in higher education research for decades. A comprehensive review by Liu et al. (2014) examined major frameworks for assessing critical thinking in higher education, identifying several key dimensions including analysis, evaluation, inference, explanation, and self-regulation. The review found that while numerous assessment instruments exist—including the California Critical Thinking Skills Test (CCTST), the Watson-Glaser Critical Thinking Appraisal, and the Cornell Critical Thinking Test—there remains considerable variation in how critical thinking is operationalized and measured across studies. This variation presents challenges for comparing findings across research contexts and for establishing clear benchmarks for critical thinking development.

Empirical research on the relationship between GAI use and critical thinking has yielded mixed findings, highlighting the importance of examining heterogeneity in use patterns. A mixed-methods study by Li et al. (2026) found that the impact of ChatGPT on critical thinking varied significantly depending on the cognitive presence phase. Cognitive presence, as defined within the Community of Inquiry framework (Garrison et al., 2001), refers to the extent to which learners are able to construct meaning through sustained communication and reflection, and it progresses through four phases: a triggering event (identifying a problem or question), exploration (searching for information and ideas), integration (connecting ideas into a coherent understanding), and resolution (applying the new understanding to solve the problem). Li et al. (2026) found that ChatGPT had positive effects on the exploration and integration phases—where AI can serve as a brainstorming partner and help synthesize diverse perspectives—but potential negative effects on the triggering event and resolution phases—where premature reliance on AI may short-circuit the learner’s own problem identification and solution verification processes. Similarly, a study examining the impact of generative AI tools on critical thinking skills among university students found that the relationship was moderated by students’ prior critical thinking abilities and their approach to using AI tools (Premkumar et al., 2024). These findings suggest that the cognitive consequences of GAI use are not uniform but depend on how students engage with these tools.

Cognitive autonomy refers to the capacity for independent thinking and self-directed learning. It involves maintaining epistemic agency—the sense that one is the author of one’s own beliefs and judgments—rather than passively accepting information from external sources. Cognitive autonomy relinquishment occurs when individuals cede their thinking to external agents, manifested as dependence, anxiety about one’s thinking ability, or uncritical acceptance of information. In the GAI context, cognitive autonomy relinquishment might appear as over-reliance on AI for answers, diminished confidence in one’s own judgment, or reduced tolerance for cognitive effort.

The concept of cognitive autonomy has roots in philosophical discussions of epistemic agency and intellectual autonomy. Kvanvig (2003) argued that epistemic agency involves not merely having true beliefs but being responsible for the formation and maintenance of one’s beliefs. This perspective suggests that cognitive autonomy is not simply about the ability to think independently but about the ownership and authorship of one’s cognitive processes. When students offload cognitive work to AI without maintaining oversight and critical evaluation, they may be relinquishing not just cognitive effort but epistemic agency—the sense that their beliefs and judgments are genuinely their own.

Research on cognitive autonomy in educational contexts has examined its relationship with academic achievement and learning outcomes. Studies have found that students with higher cognitive autonomy demonstrate greater persistence in the face of difficulty, deeper engagement with learning materials, and better transfer of knowledge to new contexts (Stefanou et al., 2004). Conversely, students who rely heavily on external sources for cognitive guidance may show reduced intrinsic motivation, lower tolerance for ambiguity, and diminished capacity for independent problem-solving. These findings suggest that cognitive autonomy relinquishment in the context of GAI use may have cascading effects on students’ broader academic development.

The relationship between critical thinking and cognitive autonomy is bidirectional and mutually reinforcing. Critical thinking skills enable students to evaluate external information sources—including AI outputs—independently and critically, thereby supporting cognitive autonomy. Conversely, cognitive autonomy provides the motivational foundation for engaging in critical thinking, as students who feel ownership over their cognitive processes are more likely to invest effort in analysis and evaluation. This bidirectional relationship suggests that interventions aimed at promoting healthy GAI use should address both the cognitive skills (critical thinking) and the dispositional factors (cognitive autonomy) that influence how students engage with AI tools.

### 2.4. Individual Factors Influencing GAI Use Patterns

The theoretical frameworks reviewed above suggest that the cognitive consequences of GAI use are not uniform but depend on individual factors that shape how students approach and engage with these tools. This section reviews research on three key individual factors: learning motivation, disciplinary background, and gender.

#### 2.4.1. Learning Motivation

Learning motivation has been extensively studied in educational psychology, with self-determination theory (SDT) providing a prominent framework for understanding different motivational orientations. SDT distinguishes between intrinsic motivation—engagement driven by inherent interest and enjoyment—and extrinsic motivation—engagement driven by external rewards or pressures (Ryan & Deci, 2020). Research has consistently shown that intrinsic motivation is associated with deeper learning, greater persistence, and better transfer of knowledge to new contexts, while extrinsic motivation is associated with surface learning, lower persistence, and poorer transfer.

In the context of technology adoption, research has examined how motivational orientations influence the ways students engage with educational technologies. A study on intrinsic motivation and ChatGPT adoption found that students with higher intrinsic motivation were more likely to use ChatGPT for exploratory learning and knowledge construction, while students with higher extrinsic motivation were more likely to use it for task completion and grade improvement (Deng & Deng, 2025). This finding suggests that motivation shapes not only whether students use GAI but how they use it—with intrinsic motivation promoting deeper, more strategic engagement and extrinsic motivation promoting more instrumental, efficiency-focused use.

The mechanism linking motivation to GAI use patterns can be understood through the lens of goal orientation theory. Students with intrinsic motivation are oriented toward learning goals—they use GAI to satisfy curiosity, build understanding, and develop competence. This orientation encourages strategic offloading, where cognitive tasks are delegated to AI in ways that support rather than replace learning. In contrast, students with extrinsic motivation are oriented toward performance goals—they use GAI to complete tasks efficiently and achieve external rewards. This orientation may encourage habitual offloading, where cognitive tasks are delegated to AI without consideration of the learning consequences.

#### 2.4.2. Disciplinary Background

Disciplinary background may influence GAI use patterns through several mechanisms. First, different disciplines have different epistemic cultures—shared assumptions about what counts as knowledge, how knowledge is produced, and what cognitive skills are valued. These epistemic cultures may shape how students approach cognitive tasks and, consequently, how they use GAI. Second, different disciplines provide different levels of exposure to and training with technology, which may influence students’ technical proficiency with GAI tools and their attitudes toward AI-mediated cognition.

Students in ICT-related disciplines (e.g., computer science, electronic engineering) may be particularly interesting cases for examining GAI use patterns. On one hand, these students typically have higher technical proficiency with digital tools and may be more skilled at using GAI’s advanced features. On the other hand, the culture of ICT disciplines often emphasizes efficiency, automation, and tool optimization—values that may inadvertently encourage habitual offloading. Research on AI literacy among ICT students has found that technical knowledge about AI systems does not automatically translate into critical awareness of AI’s cognitive and epistemic limitations (Tlili et al., 2023), potentially leaving technically proficient students vulnerable to overreliance.

#### 2.4.3. Gender Differences

Research on gender differences in technology adoption has documented varying patterns across different technologies and contexts. Studies examining generative AI adoption have found evidence of a ‘gen AI gender gap,’ with some research showing that men are more likely to have used generative AI tools than women (Aldasoro et al., 2024). However, other studies have found that when women do adopt AI tools, they may engage with them in more reflective and critical ways (Borzanović et al., 2026). These mixed findings suggest that gender differences in GAI use are complex and may be moderated by other factors such as disciplinary context and prior technology experience.

Self-efficacy theories suggest that gender differences in technology use are mediated by differences in self-efficacy beliefs, which are influenced by prior experiences and social feedback. Research on computer self-efficacy has consistently shown that women tend to report lower confidence in their ability to use technology effectively, even when actual performance levels are comparable (Hargittai & Shafer, 2006). This confidence gap may influence how female students approach GAI tools: those with lower technology self-efficacy may be more likely to accept AI outputs without verification, as they may feel less equipped to evaluate or challenge the technology’s outputs. Conversely, female students who have developed strong technology self-efficacy through positive experiences may engage with GAI in more autonomous and critical ways. These perspectives suggest that gender differences in GAI use patterns may reflect broader patterns of technology self-efficacy shaped by prior experiences and social feedback, rather than inherent gender differences in cognitive capacity.

### 2.5. Conceptual Framework

**Figure 1:**
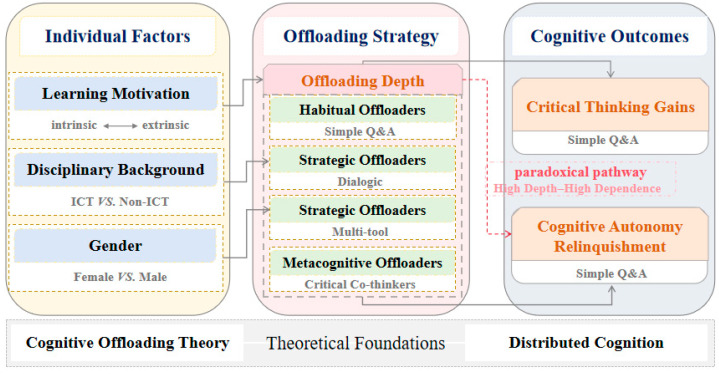
Conceptual Framework of GAI Use, Offloading Strategy, and Cognitive Outcomes.

Integrating these theoretical perspectives, we propose a conceptual framework (Figure 1) that links individual factors, offloading strategies, and cognitive outcomes. The framework posits that individual factors—learning motivation (intrinsic vs. extrinsic), disciplinary background, and gender—jointly influence the offloading strategy students adopt when using GAI. Offloading strategy, in turn, predicts two sets of cognitive outcomes: (a) perceived critical thinking gains, and (b) cognitive autonomy relinquishment.

The framework also allows for a paradoxical pathway: high offloading depth (i.e., sophisticated, multi-faceted use of GAI) does not guarantee low cognitive autonomy relinquishment. Some students may exhibit “high depth–high dependence”—a pattern in which they use GAI strategically in terms of technical features (e.g., prompt engineering, tool integration) but do so in a way that does not preserve cognitive autonomy. This paradoxical pattern is hypothesized to be more prevalent among students with extrinsic motivation, certain disciplinary backgrounds (e.g., ICT majors), and specific gender groups.

The conceptual framework integrates insights from multiple theoretical perspectives. From cognitive offloading theory, we draw the distinction between strategic and habitual offloading, and the insight that the cognitive consequences of offloading depend on metacognitive monitoring and control. From distributed cognition theory, we draw the concept of cognitive partnership and the importance of maintaining human agency in human–AI systems. From research on critical thinking and cognitive autonomy, we draw the understanding that these constructs are both cognitive and dispositional, and that they are mutually reinforcing. From research on individual differences, we draw the insight that motivational orientations, disciplinary cultures, and gender socialization shape how students approach cognitive tasks and technology use.

A key innovation of the framework is its attention to the paradoxical ‘high depth-high dependence’ pattern. This pattern challenges the intuitive assumption that sophisticated GAI use is inherently beneficial for cognitive development. Instead, the framework suggests that technical sophistication in GAI use is necessary but not sufficient for positive cognitive outcomes—students must also maintain metacognitive awareness and cognitive agency in their interactions with AI. This insight has important implications for educational practice, suggesting that interventions should focus not only on developing students’ technical skills with GAI but also on cultivating the metacognitive and motivational foundations for strategic offloading.

### 2.6. Hypotheses Development

Based on the conceptual framework and the theoretical foundations reviewed above, this study tests the following hypotheses:

**H1.**  *University students’ GAI use patterns are characterized by significant heterogeneity. Four distinct offloading profiles can be identified, ranging from habitual offloaders to metacognitive offloaders, with a subset of students exhibiting a “high offloading depth–high dependence” paradoxical pattern.*

**H2.**  *Learning motivation is the strongest predictor of both offloading strategy and cognitive outcomes. Intrinsic motivation predicts higher offloading depth, greater critical thinking gains, and lower cognitive autonomy relinquishment, while extrinsic motivation predicts the opposite pattern.*

**H3.**  *Disciplinary background and gender moderate the relationship between offloading strategy and cognitive autonomy relinquishment. ICT majors and female students are at higher risk of cognitive autonomy relinquishment, with the gender difference more pronounced among non-ICT students.*

## 3. Methods

### 3.1. Participants and Sample Characteristics

This study employed a cross-sectional survey design to investigate GAI use patterns and their associations with critical thinking and cognitive autonomy among Chinese university students. Data were collected via an online questionnaire distributed through university networks and social media platforms (primarily WeChat) between 6 February and 27 February 2025. Participants were recruited using convenience sampling, with invitations sent to students enrolled in six universities across Beijing, Shanghai, Guangzhou, and several other provinces (Hebei, Shandong, Xinjiang, etc.). A total of 353 valid responses were received after excluding incomplete submissions (response rate: 92.4% after initial screening). Table 1 presents the demographic characteristics of the sample.

The sample was predominantly undergraduate (89.8%), with a slight majority of male participants (58.4%). Disciplinary distribution covered a broad range, with STEM (non-ICT) students constituting the largest group (39.9%), followed by humanities and social sciences (26.1%), ICT majors (16.1%), and others (17.9%). While the sample was geographically concentrated in Beijing (51.8%), the remaining participants came from 14 different provinces, providing a degree of regional diversity.

### 3.2. Instruments and Variable Measurement

The questionnaire was developed based on the theoretical framework outlined in Section 2, integrating items adapted from existing scales on cognitive offloading (Risko & Gilbert, 2016), critical thinking dispositions (Nasr et al., 2025), and technology acceptance (Sergeeva et al., 2025), as well as original items designed to capture GAI-specific offloading behaviors. All items were rated on a 5-point Likert scale ranging from 1 (“strongly disagree”) to 5 (“strongly agree”). The questionnaire was initially drafted in English, then translated into Chinese using a forward-backward translation procedure by two bilingual researchers to ensure conceptual equivalence. We note that all constructs in this study were measured through self-report instruments. While self-report measures are appropriate for capturing inherently subjective constructs such as motivation and perceived dependence, they present limitations when used to assess cognitive abilities or behavioral complexity; these limitations and their implications for the interpretation of findings are discussed in detail in Section 5.5 and Section 5.6.

The measurement model comprised three core latent constructs—Offloading Depth, Critical Thinking Gains, and Cognitive Autonomy Relinquishment—along with several predictor variables. Table 2 provides an overview of the constructs, their operational definitions, example items, and reliability coefficients.

Offloading Depth was measured by five items capturing the complexity of students’ interactions with GAI. The complete set of items is as follows: (1) “I use GAI mainly for simple Q&A and translation tasks” (reverse-coded as low depth); (2) “I use GAI for multi-step reasoning tasks, such as debugging code or synthesizing literature”; (3) “I use multiple rounds of dialogue and refine my prompts to optimize GAI’s answers”; (4) “I assign GAI different roles (e.g., critic, mentor, brainstorming partner) to obtain diverse perspectives”; (5) “I integrate multiple AI tools or combine them with other software to accomplish complex tasks.” Higher scores indicate more strategic offloading; lower scores indicate more habitual offloading. We acknowledge that certain items, particularly Item 3, which refers to “optimization,” may be subject to heterogeneous interpretations among respondents. The term “optimize” implicitly assumes that participants possess a sufficiently sophisticated understanding of what optimization means within the context of GAI interaction, and responses may reflect varying levels of understanding of this concept rather than comparable levels of actual cognitive or technical engagement. This limitation is discussed further in Section 5.5.

Critical Thinking Gains were assessed with eight items covering two subdimensions: (a) efficiency gains (e.g., “Using GAI significantly saves me time in finding materials and completing assignments”) and (b) cognitive gains (e.g., “GAI helps me gain a deeper and more multidimensional understanding of complex concepts,” “GAI makes me more confident in completing creative tasks”). The efficiency subdimension comprised three items (α = 0.81); the cognitive subdimension comprised five items (α = 0.86). It should be noted that these items measure students’ perceived critical thinking gains rather than objective improvements in critical thinking ability.

Cognitive Autonomy Relinquishment was measured by five items capturing students’ sense of dependence on GAI, anxiety about their cognitive abilities, and tendency to accept AI outputs uncritically. Sample items included: “I sometimes accept GAI-generated content without critical verification” and “I feel I cannot complete assignments well without GAI assistance.” Higher scores indicate greater relinquishment of cognitive autonomy.

Learning Motivation was measured using two items adapted from self-determination theory-based scales (Ryan & Deci, 2000, 2020; Deci & Ryan, 1985): one item assessing intrinsic motivation (“I use GAI primarily because I am genuinely curious and want to deepen my understanding”) and one item assessing extrinsic motivation (“I use GAI primarily to complete assignments efficiently and achieve better grades”). The intrinsic/extrinsic motivation ratio was computed by dividing the intrinsic motivation score by the extrinsic motivation score, with higher values indicating a more intrinsic motivational orientation (M = 1.84, SD = 0.67). We acknowledge that this two-item operationalization of learning motivation is limited in its ability to capture the full complexity of motivational orientations as theorized by self-determination theory, which distinguishes among multiple subtypes of intrinsic and extrinsic motivation. This brevity was necessitated by the need to maintain a reasonable survey length while covering the multiple constructs in our framework. Future research should employ more comprehensive motivation scales to provide a more nuanced assessment of motivational profiles.

### 3.3. Data Analysis

Data analysis proceeded in four stages. First, descriptive statistics and bivariate correlations were computed for all key variables. Second, K-means cluster analysis was performed on the five offloading depth items to identify distinct user profiles; the optimal number of clusters was determined using the elbow method and silhouette coefficient. Third, multiple linear regression analyses were conducted to examine predictors of critical thinking gains and cognitive autonomy relinquishment. Fourth, hierarchical regression was used to test interaction effects, and logistic regression was employed to identify predictors of membership in the paradoxical “high depth–high dependence” group. All analyses were conducted using SPSS 27.0, with α = 0.05 as the significance threshold.

## 4. Results

### 4.1. Descriptive Statistics and Correlations

Regarding GAI usage frequency, 41.6% of students reported using GAI 1–2 times per week, 28.9% reported 3–4 times per week, 17.8% reported 5 times or more, and 7.9% reported almost never using GAI. Table 3 presents the means, standard deviations, and bivariate correlations among the core constructs and key predictors.

The mean offloading depth was 3.67 (SD = 0.92), indicating that students generally reported moderate to moderately high levels of strategic offloading. However, the relatively large standard deviation suggests substantial individual differences—a precondition for the heterogeneity analyses that follow. Critical thinking gains had a mean of 3.71 (SD = 0.88), with the efficiency subdimension (M = 3.94, SD = 0.85) scoring significantly higher than the cognitive subdimension (M = 3.48, SD = 0.96), t(352) = 6.72, *p* < 0.001. This pattern suggests that students perceive GAI as more helpful for saving time and reducing workload than for deepening conceptual understanding or enhancing analytical skills.

Cognitive autonomy relinquishment had a mean of 3.23 (SD = 0.96), with 31.2% of students agreeing or strongly agreeing that they “worry about over-reliance on GAI weakening their own thinking ability” and 22.4% agreeing or strongly agreeing that they “sometimes accept GAI-generated content without critical verification.” As shown in Table 3, offloading depth was moderately to strongly correlated with critical thinking gains (r = 0.52, *p* < 0.001) and positively correlated with cognitive autonomy relinquishment (r = 0.31, *p* < 0.001). Learning motivation exhibited the strongest correlations with both offloading depth (r = 0.48) and critical thinking gains (r = 0.44), and a negative correlation with cognitive autonomy relinquishment (r = −0.35). These preliminary associations support the theoretical expectations outlined in the conceptual framework.

### 4.2. Heterogeneity of Offloading Strategies: Cluster Analysis

To address RQ1 and test H1, a K-means cluster analysis was performed on the five offloading depth items. The optimal number of clusters was determined using the elbow method and silhouette coefficient.

**Figure 2:**
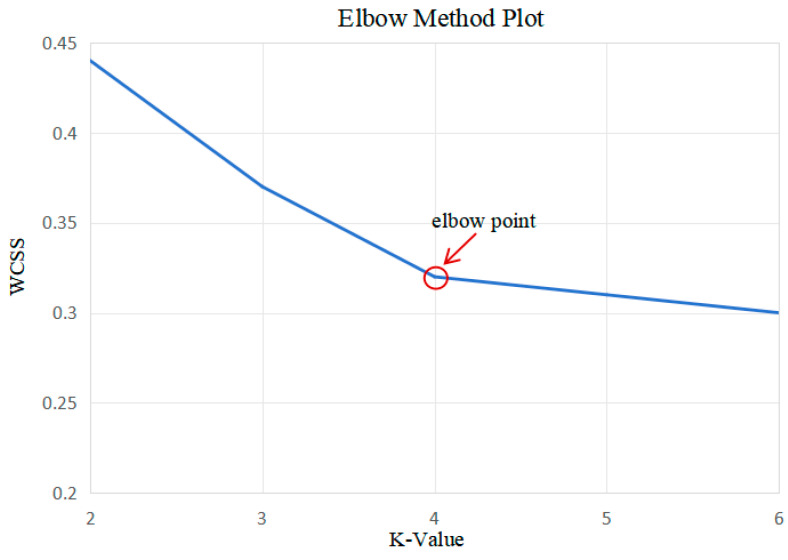
Elbow Method Plot for Determining the Optimal Number of Clusters.

As shown in Figure 2, the elbow method plot reveals a clear inflection point at k = 4, where the rate of decrease in the within-cluster sum of squares (WCSS) substantially diminishes. The silhouette coefficient further supported this solution, with values of 0.44 (k = 2), 0.37 (k = 3), 0.32 (k = 4), 0.31 (k = 5), and 0.30 (k = 6), indicating that the four-cluster solution provided the best balance between cluster separation and parsimony. Table 4 presents the cluster sizes, proportions, and defining characteristics.

A one-way ANOVA revealed significant differences among the four clusters on offloading depth, F(3, 349) = 98.42, *p* < 0.001, η^2^ = 0.46. Post hoc Tukey tests indicated that all pairwise differences were significant (*p* < 0.01) except between clusters 2 and 3 (*p* = 0.07). The hierarchical ordering (cluster 1 < cluster 2 ≈ cluster 3 < cluster 4) is consistent with a continuum from habitual to metacognitive offloading.

Importantly, the four clusters also differed significantly on critical thinking gains, F(3, 349) = 34.21, *p* < 0.001, η^2^ = 0.23, and on cognitive autonomy relinquishment, F(3, 349) = 15.67, *p* < 0.001, η^2^ = 0.12. Metacognitive offloaders (cluster 4) reported the highest critical thinking gains (M = 4.21, SD = 0.67), whereas habitual offloaders (cluster 1) reported the lowest (M = 3.12, SD = 0.84). Counterintuitively, multi-tool strategic offloaders (cluster 3) did not differ from habitual offloaders on critical thinking gains (M = 3.45, SD = 0.79 vs. M = 3.12, SD = 0.84, *p* = 0.09), despite their higher offloading depth. This suggests that technical sophistication without metacognitive engagement (i.e., using multiple tools but not critically evaluating their outputs) may not translate into perceived cognitive benefits.

Regarding cognitive autonomy relinquishment, metacognitive offloaders scored lowest (M = 2.68, SD = 0.91), indicating better preservation of cognitive autonomy, while habitual offloaders scored highest (M = 3.64, SD = 0.88). Strategic offloaders (dialogic) fell in between (M = 3.21, SD = 0.85). Notably, multi-tool strategic offloaders (cluster 3) showed elevated cognitive autonomy relinquishment (M = 3.42, SD = 0.92) relative to dialogic strategic offloaders (cluster 2), even though their offloading depth was similar. This pattern points to a paradoxical subgroup within cluster 3: those who combine high technical proficiency with high dependence.

### 4.3. Predictors of Critical Thinking Gains

To test H2 and address RQ2, a multiple linear regression was conducted with critical thinking gains as the dependent variable. Predictors included learning motivation (intrinsic/extrinsic ratio), usage frequency, disciplinary background (dummy-coded with humanities as reference), gender, and institutional resource perception. Table 5 presents the results.

Learning motivation emerged as the strongest predictor of critical thinking gains (β = 0.42, *p* < 0.001), uniquely explaining approximately 16% of the variance (semi-partial r^2^ = 0.16). Usage frequency also made a significant but substantially smaller contribution (β = 0.18, *p* = 0.003). The model explained 31% of the variance in critical thinking gains, with learning motivation accounting for the lion’s share.

To explore whether predictors differentially affected the two subdimensions of critical thinking gains (efficiency vs. cognitive), separate regressions were run (Table 6). The finding is noteworthy: usage frequency significantly predicted efficiency gains (β = 0.25, *p* = 0.002) but not cognitive gains (β = 0.12, *p* = 0.103). The result suggests that frequent use of GAI primarily enhances perceived efficiency, whereas the quality of use (captured by learning motivation) is more consequential for deeper cognitive benefits.

### 4.4. Predictors of Cognitive Autonomy Relinquishment and the Paradoxical Group

A second multiple linear regression was conducted with cognitive autonomy relinquishment as the dependent variable. In addition to the predictors used in Model 1, offloading depth was entered to examine its unique contribution. Table 7 presents the results.

Learning motivation again showed the strongest effect (β = −0.35, *p* < 0.001): higher intrinsic motivation relative to extrinsic motivation was associated with lower cognitive autonomy relinquishment. Gender also emerged as a significant predictor (β = 0.21, *p* = 0.001), with female students reporting higher relinquishment than male students. ICT majors reported higher relinquishment than non-ICT majors (β = 0.18, *p* = 0.011).

Critically, offloading depth positively predicted cognitive autonomy relinquishment (β = 0.25, *p* < 0.001), even after controlling for learning motivation and usage frequency. This counterintuitive finding—that deeper, more sophisticated offloading is associated with greater rather than lesser dependence—provides the statistical basis for the “high depth–high dependence” paradox described below. Usage frequency was not a significant predictor (β = 0.09, *p* = 0.197), reinforcing the message that how students use GAI matters more than how often. Table 8 presents the results.

To test H3’s prediction that gender differences in cognitive autonomy relinquishment vary by disciplinary background, a hierarchical regression was conducted. The interaction term was significant (β = 0.21, *p* = 0.012), and the change in R^2^ (0.03) was statistically significant. Simple slopes analysis revealed that among non-ICT students, female students reported significantly higher cognitive autonomy relinquishment than male students (b = 0.45, *p* < 0.001). Among ICT students, however, the gender difference was not significant (b = 0.09, *p* = 0.48). In other words, the higher dependence risk for female students was confined to non-ICT disciplines; within ICT, male and female students exhibited similar levels of cognitive autonomy relinquishment.

To directly examine the paradoxical pattern suggested by the positive coefficient for offloading depth in Table 7, we identified participants who scored above the sample median on both offloading depth (≥4.0) and cognitive autonomy relinquishment (≥3.5). This subgroup comprised 91 students, or 25.8% of the sample. Table 9 compares this paradoxical group with the rest of the sample on key characteristics.

Students in the paradoxical group were more likely to be female (54.9% vs. 37.0%), more likely to be ICT majors (29.7% vs. 12.2%), and had significantly lower intrinsic/extrinsic motivation ratios (1.62 vs. 1.91). Notably, their critical thinking gains did not differ from those of other students (M = 3.82 vs. 3.67, *p* = 0.156), suggesting that the cognitive benefits they derived from GAI were no greater than those of students who offloaded less deeply or less habitually—despite their sophisticated use patterns.

A logistic regression (Table 10) was conducted to identify independent predictors of membership in the paradoxical group. Learning motivation was the strongest protective factor (OR = 0.42, *p* < 0.001): a one-unit increase in the intrinsic/extrinsic ratio reduced the odds of being in the paradoxical group by 58%. Female students were more than twice as likely as male students to belong to the paradoxical group (OR = 2.15), and ICT majors were nearly three times as likely (OR = 2.89). Usage frequency did not contribute uniquely.

Taken together, these results paint a clear picture of the paradoxical group: students who use GAI with high technical sophistication (multi-tool integration, role-playing, etc.) but simultaneously experience high dependence and anxiety, and who derive no greater critical thinking gains than their peers. They are disproportionately female, disproportionately enrolled in ICT disciplines, and characterized by relatively extrinsic learning motivation.

### 4.5. Summary of Key Findings

- Four offloading profiles were identified, ranging from habitual (25.2%) to metacognitive (15.6%). Multi-tool strategic offloaders (22.1%) reported high offloading depth but did not differ from habitual offloaders on critical thinking gains.
- Learning motivation was the strongest predictor of both critical thinking gains (β = 0.42) and lower cognitive autonomy relinquishment (β = −0.35), outperforming usage frequency and demographic variables.
- Offloading depth positively predicted cognitive autonomy relinquishment (β = 0.25), revealing a paradox: deeper offloading was associated with greater dependence, not less.
- Gender and disciplinary background interacted: female students outside ICT showed higher relinquishment, but within ICT the gender gap disappeared.
- A paradoxical “high depth–high dependence” group comprised 25.8% of the sample, characterized by female gender, ICT major, and extrinsic motivation.

## 5. Discussion

### 5.1. Theoretical Implications: Beyond the “More Is Better” Assumption

The findings challenge the intuitive assumption that deeper engagement with GAI automatically translates into greater cognitive benefits. While offloading depth was positively correlated with critical thinking gains (r = 0.52), it also positively predicted cognitive autonomy relinquishment (β = 0.25) after controlling for learning motivation. This paradoxical pattern suggests that the relationship between GAI use and cognitive outcomes is not monotonic but conditional on how students approach the offloading process.

This finding resonates with emerging research on AI-mediated learning, which suggests that the cognitive consequences of technology use depend critically on the nature of human–AI interaction rather than mere exposure duration. A recent meta-analysis by Zhao et al. (2025) examining 29 experiments and quasi-experiments found that generative AI’s impact on higher-order thinking skills varied substantially depending on instructional design and learner characteristics, with effect sizes ranging from negative to strongly positive.

Theoretically, this finding extends cognitive offloading theory by highlighting the distinction between technical sophistication and metacognitive engagement. Students can be highly skilled at using GAI’s technical features (prompt engineering, multi-tool integration, role-playing) without maintaining cognitive agency in the process. This distinction aligns with Dunn and Risko’s (2016) concept of metacognitive offloading, wherein strategic decisions about what to offload are guided by learning goals rather than efficiency alone. Our results suggest that metacognitive offloading is not synonymous with sophisticated offloading; the former requires an additional layer of self-regulation that not all students possess or deploy.

From the perspective of distributed cognition theory (Hutchins, 1996), these findings illuminate the complex dynamics of cognitive work distribution in human–AI systems. Distributed cognition posits that cognitive processes are not confined to individual minds but are distributed across people, artifacts, and environments. In the context of GAI use, the critical question becomes not whether cognition is distributed—this is inevitable—but how the distribution is orchestrated. When students offload cognitive tasks to GAI, they are engaging in a form of cognitive partnership that can either enhance or diminish their cognitive capabilities depending on the nature of the partnership. Our findings suggest that without explicit metacognitive oversight, this redistribution can lead to a gradual erosion of cognitive autonomy even as technical proficiency increases.

Furthermore, our identification of four distinct user profiles contributes to the growing literature on heterogeneity in AI adoption patterns. Unlike binary characterizations of AI users as either dependent or independent, our cluster analysis reveals a more nuanced landscape. The ‘critical co-thinkers’ profile (15.6% of the sample) demonstrates that sophisticated GAI use can coexist with maintained cognitive autonomy when guided by metacognitive awareness. This finding aligns with earlier work by Lowther et al. (2008) on technology integration and cognitive offload instruction, which showed that when GAI is framed as a metacognitive tool rather than an answer generator, students can achieve significant gains in critical thinking while maintaining cognitive agency. The theoretical implication is clear: the cognitive consequences of GAI use are not determined by the technology itself but by the cognitive stance that learners adopt toward it.

An important caveat regarding the ‘critical co-thinkers’ profile is that the cross-sectional design of our study cannot determine whether these students developed metacognitive engagement patterns through their GAI use or whether they possessed higher baseline cognitive abilities that enabled them to engage with GAI in this way from the outset. It is plausible that the 15.6% of students classified as critical co-thinkers are simply those with stronger pre-existing critical thinking skills and greater metacognitive awareness—characteristics that would lead them to approach any cognitive tool, including GAI, in a more strategic and autonomous manner. This possibility raises a crucial question for intervention design: can metacognitive training enable the remaining 84.4% of students to adopt similar patterns of GAI engagement, or are there stable individual differences that limit the effectiveness of such interventions? While our data cannot definitively answer this question, the strong predictive role of learning motivation—a modifiable factor—in distinguishing critical co-thinkers from other profiles suggests that intervention is at least partially feasible. Future research using pre-post designs with randomized assignment to metacognitive training conditions is needed to determine the extent to which the critical co-thinker profile can be cultivated through educational intervention.

However, the interpretation of these findings must be tempered by an important methodological consideration. Because all constructs in this study were measured through self-report instruments, the observed relationships may be partially inflated by common-method variance (Podsakoff et al., 2003), and the “critical thinking gains” we report should be understood as students’ perceived gains rather than objective improvements in critical thinking ability. From this perspective, the apparent success of the critical co-thinkers may reflect not only their superior metacognitive engagement but also their greater metacognitive awareness—that is, their ability to accurately perceive and report their own cognitive processes. Conversely, the paradoxical group’s reported combination of high technical sophistication and high dependence may partly reflect a response style characterized by self-enhancement on technical dimensions combined with self-critical awareness on dependence dimensions, rather than a genuine cognitive-behavioral pattern. These alternative interpretations, grounded in the literature on self-assessment accuracy (Dunning et al., 2003) and common-method bias (Podsakoff et al., 2003), do not invalidate our findings but highlight the need for caution in drawing strong conclusions about the cognitive mechanisms underlying the identified profiles. Future research employing objective performance-based measures of critical thinking and behavioral measures of GAI use is needed to disentangle genuine cognitive patterns from perceptual biases.

### 5.2. The Centrality of Learning Motivation

Learning motivation emerged as the most powerful predictor across all analyses. Students with higher intrinsic relative to extrinsic motivation reported greater offloading depth, higher critical thinking gains, and lower cognitive autonomy relinquishment. They were also less likely to belong to the paradoxical “high depth–high dependence” group (OR = 0.42). This pattern is consistent with self-determination theory (Deci & Ryan, 2000), which posits that intrinsic motivation fosters deeper engagement, greater persistence, and more meaningful learning outcomes.

Prior research on intrinsic motivation and technology adoption has shown that students driven by curiosity and intrinsic interest are more likely to engage with digital tools for exploratory learning rather than mere task completion, mirroring our findings on the relationship between motivation and offloading patterns.

The mechanism linking motivation to offloading outcomes can be understood through the lens of goal orientation. Intrinsically motivated students use GAI as a tool for exploration and understanding—they ask questions to satisfy curiosity, seek explanations to build conceptual frameworks, and engage in dialogue to refine their thinking. Extrinsically motivated students, by contrast, use GAI as a means to external ends—completing assignments, obtaining grades, meeting deadlines. When the goal is task completion rather than understanding, the path of least resistance (accepting AI outputs uncritically) becomes more attractive than the cognitively demanding path of verification and synthesis.

This motivational mechanism can be further elaborated through the framework of self-determination theory, which identifies three basic psychological needs: autonomy, competence, and relatedness (Ryan & Deci, 2020). Intrinsically motivated learners are more likely to experience GAI use as autonomy-supportive—they choose to engage with AI as a cognitive partner rather than feeling compelled to use it for external rewards. This sense of volitional engagement may protect against the erosion of cognitive autonomy that characterizes the paradoxical offloading pattern. Conversely, extrinsically motivated learners may experience GAI as controlling rather than supporting, leading to a more passive and dependent relationship with the technology. Recent research on digital learning from a self-determination theory perspective (Chiu, 2024) supports this interpretation, showing that technology integration that supports psychological needs leads to deeper learning outcomes, while technology use driven by external contingencies tends to produce surface-level engagement.

The practical significance of motivation as a predictor is underscored by its effect size relative to other variables. With a standardized coefficient of β = 0.42 for critical thinking gains, learning motivation outperformed usage frequency, technical proficiency, and even offloading depth in predicting positive cognitive outcomes. This finding has important implications for intervention design: rather than focusing primarily on technical training or usage guidelines, educators may need to address the motivational foundations of student engagement with GAI. Interventions that foster intrinsic motivation—such as emphasizing the inherent interest value of learning tasks, providing autonomy-supportive choices, and connecting academic work to students’ personal goals—may be more effective in promoting healthy GAI use patterns than technical skill development alone.

### 5.3. The Price of Offloading: When Strategic Offloading Tips into Habitual Dependence

The identification of a paradoxical “high depth–high dependence” user profile (25.8% of the sample) represents one of the most theoretically significant findings of this study. These students reported sophisticated technical engagement with GAI—indicating that they perceived themselves as using multiple tools, employing role-playing prompts, and integrating AI outputs into complex workflows—yet simultaneously reported high levels of cognitive dependence and anxiety, and derived no greater critical thinking benefits than less sophisticated users. This pattern challenges the assumption that technical proficiency in AI use automatically translates into cognitive benefits. It is important to note, however, that the evidence for sophisticated engagement is based entirely on self-reported questionnaire responses; the study does not provide objective evidence that these students actually engaged with GAI in the sophisticated ways they described, but rather shows that they perceived or reported themselves as doing so.

This paradoxical pattern can be understood through the lens of cognitive load theory and the distinction between germane and extraneous cognitive load (Sweller, 2020). When students invest cognitive resources in mastering technical aspects of GAI use—learning prompt engineering, integrating multiple tools, developing efficient workflows—these resources become unavailable for the deeper cognitive processing that supports critical thinking development. The technical sophistication that characterizes the paradoxical group may thus represent a form of ‘cognitive misallocation,’ where students become expert at using the tool but invest insufficient effort in the metacognitive processes that would make tool use cognitively productive.

#### 5.3.1. ICT Students: High Technical Skill, High Dependence Risk

ICT majors (computer science, electronic engineering, etc.) were overrepresented in the paradoxical “high depth–high dependence” group (OR = 2.89). They reported high technical proficiency in using GAI—claiming to integrate multiple tools, write sophisticated prompts, and automate complex workflows—yet they also reported higher cognitive autonomy relinquishment than non-ICT students (β = 0.18). This finding challenges the intuitive assumption that technical skill automatically confers cognitive resilience. Again, we emphasize that these characterizations are based on students’ self-perceptions of their technical engagement rather than on objective behavioral measures. Why might ICT students be at greater risk? Several explanations are plausible.

First, ICT students’ deeper understanding of how GAI models work (e.g., transformer architectures, training data biases) may paradoxically increase their trust in AI outputs. Knowing that GAI is “just a statistical pattern matcher” might not reduce reliance; it could instead lead to a form of “informed fatalism”—accepting outputs because one understands the constraints but feels powerless to overcome them.

Second, ICT curricula often emphasize efficiency, automation, and tool mastery as professional values. These values, while professionally relevant, may inadvertently cultivate a mindset that prioritizes output optimization over cognitive engagement. When students are trained to seek the most efficient solution to technical problems, this heuristic may generalize to academic learning contexts where efficiency is not the appropriate metric. The professional culture of ICT fields—which celebrates automation and the replacement of human labor with computational solutions—may normalize cognitive offloading in ways that students in other disciplines do not experience. Recent research on AI literacy in higher education (Tlili et al., 2023) suggests that technical knowledge about AI systems does not automatically translate into critical awareness of AI’s cognitive and epistemic limitations, potentially leaving technically proficient students vulnerable to overreliance.

Third, ICT students may face unique pressures related to the rapid evolution of their field. The constant emergence of new tools, frameworks, and technologies creates a context where staying current requires continuous learning and adaptation. In this environment, GAI may appear as an essential tool for managing information overload and maintaining professional relevance. However, this instrumental reliance on AI for knowledge management may inadvertently undermine the development of deeper conceptual understanding that would enable students to evaluate and integrate new information independently. The tension between using AI as a professional tool and maintaining cognitive autonomy represents a challenge that ICT education has yet to adequately address.

However, an alternative interpretation, grounded in the literature on common-method variance (Podsakoff et al., 2003), must be acknowledged: the higher dependence reported by ICT students may partially reflect a heightened critical self-awareness of their reliance on technology, rather than a purely negative outcome. Without objective behavioral data, we cannot distinguish between these possibilities.

#### 5.3.2. Gender Differences in Offloading Styles

Female students reported higher cognitive autonomy relinquishment than male students (β = 0.21), and were more than twice as likely to belong to the paradoxical group (OR = 2.15). However, this gender difference was moderated by disciplinary background: among non-ICT students, females showed significantly higher relinquishment; among ICT students, the gender gap disappeared.

This interaction effect is intriguing and warrants careful interpretation. One possibility is that ICT disciplines attract or cultivate a particular profile of female students—those with higher technical confidence and greater familiarity with computational tools—who are consequently less susceptible to cognitive dependence on GAI. Another possibility is that the male-dominated environment of ICT programs creates social pressure for female students to demonstrate competence, leading them to maintain greater cognitive vigilance when using AI tools.

These findings contribute to an emerging literature on gender differences in AI adoption and use patterns. Recent research has documented a ‘gen AI gender gap’ in professional contexts, with studies showing that men are more likely than women to have used generative AI tools (Aldasoro et al., 2024). However, our findings suggest a more complex picture in educational contexts: while female students in our sample showed higher cognitive dependence, they did not show lower usage frequency or technical sophistication.

The disappearance of gender differences among ICT students is particularly noteworthy and may reflect the operation of selection effects, socialization processes, or both. Female students who choose ICT majors may possess higher baseline levels of technical self-efficacy that protect against cognitive dependence, or the process of succeeding in a male-dominated field may cultivate cognitive resilience. Alternatively, the competitive environment of ICT programs may create similar pressures for all students, regardless of gender, to demonstrate independent problem-solving capabilities. Future research should examine these mechanisms more closely, as understanding why the gender gap disappears in ICT contexts could inform interventions to reduce cognitive dependence in other disciplines.

While this pattern aligns with previous research on technology self-efficacy, the self-reported nature of our data raises the possibility of shared method variance inflating the relationship between gender and perceived dependence. Future research with performance-based measures is essential for validation.

### 5.4. Practical Implications for Education

The findings have several implications for educational practice. First, interventions should focus less on technical training (how to write better prompts, how to integrate multiple tools) and more on metacognitive development (how to decide what to offload, how to maintain cognitive agency, how to critically evaluate AI outputs). Technical skill without metacognitive regulation may inadvertently increase dependence risk.

This recommendation aligns with emerging best practices in AI literacy education. Researchers have argued that AI literacy should encompass not only technical knowledge about how AI systems work but also critical awareness of AI’s epistemic limitations and the metacognitive skills needed to maintain cognitive autonomy (Long & Magerko, 2020). Our findings provide empirical support for this expanded conception of AI literacy by demonstrating that technical proficiency alone does not protect against—and may even increase the risk of—cognitive dependence. Educational interventions should explicitly teach students to ask metacognitive questions when using GAI: What cognitive work am I offloading? Why am I choosing to offload this particular task? How will I verify and integrate the AI’s output? What am I learning through this interaction?

Second, educators should pay particular attention to students in high-risk categories: those with predominantly extrinsic motivation, ICT majors, and female students in non-ICT disciplines. These students may benefit from targeted interventions that foster intrinsic learning goals and provide structured opportunities for independent cognitive work.

For ICT students specifically, interventions should address the potential mismatch between professional values of efficiency and academic goals of deep learning. Educators might explicitly discuss when efficiency is appropriate (e.g., routine coding tasks, information retrieval) and when cognitive engagement is essential (e.g., conceptual understanding, creative problem-solving). Case studies illustrating the limitations of AI-generated solutions in professional contexts could help students recognize the continued importance of independent cognitive capabilities. Additionally, incorporating reflective exercises that prompt students to articulate what they have learned through AI-assisted work—versus what they have merely produced—may help maintain awareness of the distinction between output and understanding.

Third, the finding that usage frequency predicted efficiency gains but not cognitive gains suggests that simply encouraging more GAI use is unlikely to yield deeper learning benefits. Quality of use—guided by learning motivation and metacognitive awareness—matters more than quantity of use. Educational policies that restrict or promote GAI use should consider not just whether students use GAI, but how they use it.

This finding has important implications for institutional policies on GAI use. Blanket prohibitions may be counterproductive, preventing students from developing the skills needed for effective human–AI collaboration in their future careers. Conversely, uncritical promotion of GAI adoption may inadvertently encourage the development of dependent use patterns. A more nuanced approach is needed—one that recognizes GAI as a powerful cognitive tool while explicitly teaching students to maintain cognitive agency in their interactions with it. As suggested by recent research on AI in education (Mustafa et al., 2024), the goal should be to develop students’ capacity for ‘critical AI literacy’—the ability to use AI tools strategically while maintaining the metacognitive awareness needed to evaluate, verify, and integrate AI outputs into one’s own cognitive work.

### 5.5. Limitations and Future Directions

Several limitations should be acknowledged. First, the cross-sectional design precludes causal inference. While we used regression language (“predicts”), the associations observed may reflect reverse causation or third-variable confounds. Longitudinal studies are needed to examine whether GAI use patterns predict changes in critical thinking and cognitive autonomy over time.

The cross-sectional nature of our data is particularly limiting for understanding the dynamics of cognitive dependence development. It remains unclear whether the paradoxical offloading pattern we identified represents a stable user profile or a transitional stage in students’ relationship with GAI. Longitudinal research could examine whether students naturally progress from dependent to autonomous use patterns as they gain experience, or whether early patterns of use become entrenched and resistant to change. Such research would have important implications for the timing and content of educational interventions. We specifically recommend longitudinal studies that track students’ AI-use behaviors and cognitive outcomes over an academic semester or longer, using experience sampling methods to capture within-person changes in offloading patterns. Additionally, experimental designs that manipulate the type of GAI use (e.g., metacognitive guidance vs. free use) and measure both perceived and objective critical thinking outcomes before and after the intervention would provide stronger evidence for causal claims.

Second, the sample was predominantly undergraduate (89.8%) and geographically concentrated in Beijing (51.8%), limiting generalizability. Future research should examine whether the observed patterns hold among graduate students, students in different regions, and students in different educational systems. The concentration of our sample in Beijing raises questions about the influence of regional and institutional factors on GAI use patterns. Beijing’s universities may have different technological infrastructure, academic cultures, and AI policies compared to institutions in other regions. Additionally, the competitive academic environment in Beijing may shape students’ motivational orientations in ways that influence their GAI use. Comparative research across diverse institutional and regional contexts would help establish the robustness of our findings and identify contextual factors that moderate the relationship between GAI use and cognitive outcomes.

Third, the measures relied on self-report, which may be subject to social desirability bias, recall limitations, and common-method variance. The exclusive reliance on self-reported measures collected through the same instrument and at the same moment in time introduces a substantial risk of common-method variance (Podsakoff et al., 2003), which may inflate the observed correlations between constructs. The relationships identified between variables could be partially explained by common-method bias rather than genuine theoretical relationships. This concern is particularly acute because the study treats constructs that differ in their ontological status—subjective perceptions (e.g., motivation, anxiety) and cognitive competencies (e.g., critical thinking)—as methodologically equivalent through the exclusive use of self-report instruments.

Self-report measures of cognitive autonomy relinquishment may be particularly vulnerable to bias, as students may be reluctant to acknowledge dependence on AI tools. More fundamentally, the study may not be measuring critical thinking itself, but rather students’ perceived critical thinking ability. Similarly, “offloading depth” may reflect students’ subjective interpretations of what constitutes complex AI usage, rather than objectively observable cognitive practices. The distinction between perceived and demonstrated cognition is critical because the interpretation of the results changes substantially depending on whether the study is understood as measuring perceived cognition or demonstrated cognition. Future research should incorporate behavioral measures, such as tracking the types of tasks students choose to offload versus complete independently, the degree to which students modify AI-generated outputs, and the time spent in verification activities. Objective assessments of critical thinking, such as the California Critical Thinking Skills Test or the Watson-Glaser Critical Thinking Appraisal, would provide more reliable outcome measures than perceived gains. Additionally, think-aloud protocols during GAI-assisted tasks could provide rich data on students’ metacognitive processes and decision-making during offloading.

Fourth, the study focused on perceived critical thinking gains rather than objective improvements in critical thinking ability. While perceived gains are meaningful—they may influence students’ continued use of GAI and their academic self-concept—they may not correspond to actual skill development. As noted in Section 5.1, this limitation is not merely incidental but is central to the interpretation of the study’s findings. When we report that certain student profiles show higher “critical thinking gains,” we are reporting that these students perceive themselves as having gained more in critical thinking—not that they have objectively improved. This distinction is particularly important for the interpretation of the “high depth–high dependence” profile: these students report sophisticated GAI use but also high dependence, and it is possible that both reports reflect a common underlying factor (e.g., a tendency toward self-enhancement or a general response style) rather than genuine cognitive and behavioral patterns. Future research should incorporate standardized critical thinking assessments to examine whether perceived gains translate into measurable improvements.

Finally, future research should examine the role of instructional context in shaping GAI use patterns. Our study focused on individual differences in motivation, technical skill, and demographic characteristics, but the way GAI is integrated into courses—whether it is encouraged, discouraged, or guided with specific protocols—likely influences how students approach cognitive offloading. Research comparing different pedagogical approaches to GAI integration could identify best practices for promoting metacognitive offloading while preventing cognitive dependence. Additionally, qualitative research exploring students’ lived experiences of GAI use could provide deeper insight into the psychological processes underlying the patterns we observed quantitatively. Most importantly, future research should employ multi-method designs that combine self-report measures with objective performance-based assessments, behavioral log data, and qualitative methods to provide a more comprehensive and valid assessment of the cognitive consequences of GAI use.

### 5.6. Methodological Reflection: The Distinction Between Perceived and Demonstrated Cognition

Building on the methodological considerations integrated into the interpretation of our findings in Section 5.1, this section provides an extended discussion of the distinction between perceived and demonstrated cognition and its broader implications. Throughout this manuscript, we have used terms such as “perceived critical thinking gains” and “self-reported offloading depth” to emphasize that our measures capture students’ perceptions of their cognitive behaviors and abilities rather than objective evidence of actual performance. This distinction is not merely semantic; it fundamentally affects how the results should be interpreted and what conclusions can legitimately be drawn.

Critical thinking is generally understood as a performance-based competency that requires objective assessment through tasks, problem-solving activities, or validated performance measures (Facione, 1990; Liu et al., 2014). When we measure critical thinking through self-report, we are assessing a related but distinct construct—students’ beliefs about their critical thinking abilities and their perceptions of whether GAI use has improved those abilities. These perceptions may be influenced by a variety of factors unrelated to actual critical thinking performance, including social desirability, self-enhancement biases, and the difficulty of accurately evaluating one’s own cognitive processes (Dunning et al., 2003).

Similarly, “offloading depth” as measured in this study reflects students’ subjective interpretations of what constitutes complex or sophisticated AI usage. The item referring to “prompts to optimize GAI’s answers,” for example, implicitly assumes that participants share a common understanding of what “optimization” means in the context of GAI interaction. In reality, responses may reflect heterogeneous interpretations of this concept, with some students interpreting “optimization” as simply rephrasing a question and others understanding it as involving sophisticated prompt engineering techniques. This variability in interpretation means that the same score on the offloading depth scale may reflect qualitatively different levels of actual cognitive engagement with GAI.

The exclusive reliance on self-reported measures collected through the same instrument at the same time also introduces the risk of common-method variance (Podsakoff et al., 2003). Common-method variance refers to the variance that is attributable to the measurement method rather than to the constructs the measures represent, and it can inflate observed correlations between variables. In our study, the moderate to strong correlation between offloading depth and perceived critical thinking gains (r = 0.52) may be partially inflated by common-method bias, as both constructs were measured using the same Likert-scale format in the same questionnaire administration. Similarly, the positive correlation between offloading depth and cognitive autonomy relinquishment (r = 0.31) could reflect shared method variance rather than a genuine theoretical relationship.

These considerations suggest that our findings should be interpreted as mapping the landscape of students’ self-perceived cognitive relationship with GAI, rather than as providing objective evidence of actual cognitive outcomes. This framing does not diminish the value of the study—students’ perceptions of their cognitive behaviors and abilities are themselves important phenomena that influence their future behavior, academic choices, and relationship with technology. However, it does mean that the conclusions drawn from this study must be carefully qualified. When we identify a “high depth–high dependence” profile, we are identifying a group of students who perceive themselves as using GAI in sophisticated ways while also perceiving themselves as cognitively dependent on it. Whether these perceptions correspond to objective reality remains an open question that future research must address through multi-method designs combining self-report with behavioral and performance-based measures.

## 6. Conclusions

This study investigated the heterogeneity of critical thinking outcomes among Chinese university students using generative artificial intelligence. Drawing upon cognitive offloading theory and distributed cognition theory, we identified four distinct user profiles, examined predictors of critical thinking gains and cognitive autonomy relinquishment, and documented a paradoxical “high depth–high dependence” pattern that challenges the assumption that sophisticated GAI use automatically yields cognitive benefits.

The findings underscore the centrality of learning motivation in shaping how students engage with GAI and what they derive from it. Intrinsically motivated students tend to use GAI as a thinking partner, maintaining cognitive agency while benefiting from AI assistance. Extrinsically motivated students are more likely to use GAI as a thinking replacement, offloading cognitive work without maintaining the metacognitive oversight necessary for genuine learning.

The paradoxical group—students who perceive themselves as combining high technical sophistication with high cognitive dependence—represents a particularly concerning pattern. These students report having mastered the technical aspects of GAI use but have not developed the metacognitive habits that would allow them to benefit cognitively from this sophistication. They are disproportionately female, disproportionately enrolled in ICT disciplines, and characterized by extrinsic motivation.

As GAI tools become increasingly embedded in educational contexts, the question of how to ensure that cognitive offloading enhances rather than undermines critical thinking becomes ever more pressing. This study suggests that the answer lies not in restricting access to GAI or in promoting technical skill development, but in cultivating the metacognitive capacities and intrinsic motivation that enable students to maintain cognitive agency in an age of artificial intelligence. Because all constructs were measured through self-report, these conclusions should be understood as reflecting students’ perceptions of their cognitive behaviors and outcomes; future research employing multi-method designs that combine self-report with objective performance-based assessments is essential to validate and extend the patterns identified in this study.

## Figures and Tables

**Table 1:** Demographic Characteristics of the Sample (*n* = 353).

Characteristic	Category	*n*	%
Gender	Male	206	58.4
	Female	147	41.6
Education level	Undergraduate	317	89.8
	Master’s	34	9.6
	Doctoral	2	0.6
Academic discipline	STEM (non-ICT)	141	39.9
	Humanities & Social Sciences	92	26.1
	ICT (Computer Science, EE, etc.)	57	16.1
	Others (Business, Arts, etc.)	63	17.9
Geographic region	Beijing	183	51.8
	Other provinces	170	48.2

Note: STEM = Science, Technology, Engineering, Mathematics; ICT = Information and Communication Technology.

**Table 2:** Constructs, Operational Definitions, and Sample Items.

Construct	Operational Definition	Sample Item	No. of Items	Cronbach’s α
Offloading Depth	The sophistication and intentionality of GAI use, reflecting strategic vs. habitual offloading	“I use multiple rounds of dialogue and refine my prompts to optimize GAI’s answers”	5	0.85
Critical Thinking Gains	Perceived improvement in analytical, evaluative, and creative thinking as a result of GAI use	“Using GAI helps me gain a deeper and more multidimensional understanding of complex concepts”	8	0.88
Cognitive Autonomy Relinquishment	Extent to which students cede independent thinking to GAI, manifested as dependence, anxiety, or uncritical acceptance	“I worry that over-reliance on GAI will weaken my own thinking ability”	5	0.82

Note: All items were measured on a 5-point Likert scale (1 = strongly disagree, 5 = strongly agree).

**Table 3:** Means, Standard Deviations, and Correlations Among Key Variables.

Variable	M	SD	1	2	3	4	5
1. Offloading Depth	3.67	0.92	—				
2. Critical Thinking Gains	3.71	0.88	0.52 ***	—			
3. Cognitive Autonomy Relinquishment	3.23	0.96	0.31 ***	0.08	—		
4. Learning Motivation (intrinsic/extrinsic ratio)	1.84	0.67	0.48 ***	0.44 ***	−0.35 ***	—	
5. Usage Frequency	2.56	0.89	0.37 ***	0.28 ***	0.21 **	0.15 *	—

Note: N = 353. Learning motivation ratio is based on two items (intrinsic/extrinsic). * *p* < 0.05, ** *p* < 0.01, *** *p* < 0.001.

**Table 4:** Four-Cluster Solution of Offloading Strategies.

Cluster	Label	*n*	%	Defining Characteristics
1	Habitual Offloaders (Simple Q&A)	89	25.2	Use GAI primarily for simple knowledge retrieval and translation; rarely engage in multi-round dialogue, role-playing, or multi-tool integration
2	Strategic Offloaders (Dialogic)	131	37.1	Employ multi-round dialogue and prompt refinement to deepen answers; do not typically use role-playing or integrate multiple tools
3	Strategic Offloaders (Multi-tool)	78	22.1	Skilled at integrating multiple AI tools or combining them with other software to complete complex projects
4	Metacognitive Offloaders (Critical Co-thinkers)	55	15.6	Use GAI strategically; assign it different roles (e.g., critic, mentor, brainstorming partner) to challenge and extend their own thinking

**Table 5:** Regression Analysis Predicting Critical Thinking Gains.

Predictor	β	SE	t	*p*	95% CI
Learning Motivation (intrinsic/extrinsic ratio)	0.42	0.05	8.40	<0.001	[0.32, 0.52]
Usage Frequency	0.18	0.06	3.00	0.003	[0.06, 0.30]
Disciplinary Background (ICT vs. Humanities)	0.09	0.07	1.29	0.197	[−0.05, 0.23]
Disciplinary Background (STEM vs. Humanities)	0.07	0.07	1.00	0.318	[−0.07, 0.21]
Gender (Female vs. Male)	0.06	0.06	1.00	0.317	[−0.06, 0.18]
Institutional Resource Perception	0.08	0.06	1.33	0.184	[−0.04, 0.20]

Note: R^2^ = 0.31, adjusted R^2^ = 0.29, F(6, 345) = 21.83, *p* < 0.001. β = standardized coefficient. CI = confidence interval.

**Table 6:** Regression Results for Efficiency Gains and Cognitive Gains.

Predictor	Efficiency Gains (β)	Cognitive Gains (β)
Learning Motivation	0.35 ***	0.38 ***
Usage Frequency	0.25 **	0.12
Disciplinary Background (ICT vs. Humanities)	0.10	0.08
Gender	0.05	0.07
Institutional Resource Perception	0.06	0.09

Note: *** *p* < 0.001, ** *p* < 0.01, *p* < 0.05.

**Table 7:** Regression Analysis Predicting Cognitive Autonomy Relinquishment.

Predictor	β	SE	t	*p*	95% CI
Learning Motivation (intrinsic/extrinsic ratio)	−0.35	0.06	−5.83	<0.001	[−0.47, −0.23]
Gender (Female vs. Male)	0.21	0.06	3.50	0.001	[0.09, 0.33]
Disciplinary Background (ICT vs. Non-ICT)	0.18	0.07	2.57	0.011	[0.04, 0.32]
Offloading Depth	0.25	0.06	4.17	<0.001	[0.13, 0.37]
Usage Frequency	0.09	0.07	1.29	0.197	[−0.05, 0.23]
Institutional Resource Perception	−0.05	0.06	−0.83	0.407	[−0.17, 0.07]

Note: R^2^ = 0.29, adjusted R^2^ = 0.27, F(6, 345) = 20.05, *p* < 0.001. β = standardized coefficient. Non-ICT includes humanities, STEM, and other disciplines.

**Table 8:** Hierarchical Regression for Gender × ICT Interaction.

Step	Predictor	β	ΔR^2^	F Change	*p*
Step 1	Gender	0.19 **	0.08	15.24	<0.001
	ICT (vs. non-ICT)	0.15 *			
Step 2	Gender	0.12	0.03	6.32	0.012
	ICT	0.07			
	Gender × ICT	0.21 *			

Note: ** *p* < 0.01, * *p* < 0.05.

**Table 9:** Characteristics of the Paradoxical “High Depth–High Dependence” Group.

Characteristic	Paradoxical Group (*n* = 91)	Others (*n* = 262)	Test Statistic	*p*
Offloading Depth (M, SD)	4.32 (0.28)	3.45 (0.89)	t(351) = 9.12	<0.001
Cognitive Autonomy Relinquishment (M, SD)	4.01 (0.35)	2.98 (0.92)	t(351) = 10.45	<0.001
Critical Thinking Gains (M, SD)	3.82 (0.81)	3.67 (0.90)	t(351) = 1.42	0.156
Learning Motivation Ratio (M, SD)	1.62 (0.58)	1.91 (0.68)	t(351) = −3.67	<0.001
Proportion Female	54.9%	37.0%	χ^2^(1) = 8.94	0.003
Proportion ICT Major	29.7%	12.2%	χ^2^(1) = 15.21	<0.001

**Table 10:** Logistic Regression Predicting Paradoxical Group Membership.

Predictor	OR	95% CI	*p*
Learning Motivation Ratio	0.42	[0.28, 0.63]	<0.001
Gender (Female vs. Male)	2.15	[1.28, 3.61]	0.004
ICT Major (vs. non-ICT)	2.89	[1.58, 5.29]	0.001
Usage Frequency	1.21	[0.89, 1.64]	0.223

Note: OR = odds ratio. CI = confidence interval.

## Data Availability

The data supporting the findings of this study are available from the corresponding author upon reasonable request.
